# Does reverse transport of dopamine play a role in autism?

**DOI:** 10.1016/j.ebiom.2015.01.012

**Published:** 2015-01-20

**Authors:** Enzo Emanuele

**Affiliations:** Living Research s.a.s., Via Monte Grappa, 13, I-27038 Robbio, PV, Italy

Dysfunction in dopaminergic signaling may be an underlying cause of different neuropsychiatric disorders, including schizophrenia, bipolar disorder, attention-deficit hyperactivity disorder (ADHD), and autism (Money & Stanwood, 2013). The dopamine (DA) transporter (DAT) plays a critical role in regulating the strength of dopaminergic tone by clearing extracellular DA (Vaughan & Foster, 2013). Interestingly, DAT is the site of action for psychostimulants such as amphetamine (AMPH), which is thought to elevate extracellular DA levels by competitively inhibiting DA uptake, ultimately causing reverse transport of DA (DA efflux) (Robertson et al., 2009). Growing evidence indicates a genetic link between DAT and autism (Bowton et al., 2014, Hamilton et al., 2013). The work by Cartier et al. (in press) in this issue of *EBioMedicine* has studied the functional consequences of a nonsynonymous genetic variant in the human DAT gene (SLC6A3) which converts Arg51 to tryptophan (SLC6A3 R51W) in a family with autism. Moreover, the authors showed that another autism-causing rare variant in the syntaxin 1A gene (STX1A), which converts Arg26 to glutamine (STX1A R26Q), disrupts the molecular mechanisms of reverse transport of DA. Based on these results, they concluded that rare variants causing a significant inhibition of reverse transport of DA may play a pathogenic role in autism. While it has been known for many years that reverse transport of DA may be involved in the maintenance of an optimal range of DA levels (Leviel, 2001), its exact role in the pathogenesis of neuropsychiatric disorders remains to be determined. The molecular analyses provided in the paper by Cartier et al. (in press) elegantly demonstrate that alterations in reverse transport of DA may be involved in the pathogenesis of autism symptoms at least in the specific pedigrees. However, the question as to whether such rare genetic variants affecting the reverse transport of DA might influence the risk of common, sporadic forms of autism remains open. It is now widely accepted that rare genetic variants could play an important role in susceptibility to common diseases. In light of Cartier et al.'s results, major resequencing efforts of the SLC6A3 and STX1A genes should be undertaken in large cohorts of individuals with autism, with significant insights into disease biology likely from these results.

Another interesting implication of the work by Cartier and colleagues lies in unraveling the therapeutic potential of DA reverse transport in autism. Starting from the observation that rare autism-causing variants are associated with inhibition of reverse transport of DA, it could be hypothesized that psychostimulants may serve as a potential therapeutic aid in autism. Notably, not only have psychostimulants been suggested to be effective for ADHD-like symptoms in autism spectrum disorder individuals, but the prevalence rates of their use in autism are also strikingly increasing (Dalsgaard et al., 2013). It should be noted, however, that AMPH (which is known to cause reverse transport of DA) has been also shown to elicit autism-like symptoms including stereotyped behaviors and social impairments in animal models (Moy et al., 2013, Moy et al., 2014). Based on this evidence, the conclusion that increasing the reverse transport of DA could represent a novel therapeutic target in autism per se (and not for the ADHD-like symptoms in autism) is probably too premature. However, it is becoming increasingly clear that the reverse transport of DA could be one of the key mechanisms involved in the fine-tuning of DA levels in the synapse. In this scenario, the work by Cartier and colleagues is relevant in unraveling how disruption of such synaptic dopaminergic fine-tuning could ultimately result in autism.

## Author Contribution

EE was the sole contributor to this paper.

## Funding

This paper did not receive any funding.

## Conflicts of Interest

EE declared that he has no conflicts of interest.
